# Optimising rhinoplasty outcomes in patients with thick skin

**DOI:** 10.1016/j.jpra.2025.05.010

**Published:** 2025-05-30

**Authors:** Catrin Wigley, Jonathan Sutton, Nicholas Eynon-Lewis, Rajan Uppal

**Affiliations:** a Royal Free Hospitals, London, UK; b Portsmouth Hospitals NHS Trust, UK; c Weymouth Street Hospital, London, UK

**Keywords:** Rhinoplasty, Skin thickness, Cosmetic surgery, Chemical peel

## Abstract

**Background:**

Performing rhinoplasty in patients with a thick skin often presents as a challenge. This article describes an assessment tool to (1) classify patients according to the *thickness* of their skin and (2) to demonstrate the *clinical application* of this classification for the benefit of patients before Rhinoplasty.

**Methods:**

166 patients underwent open rhinoplasty and were graded according to the Nasal Skin Thickness Scale (NSTS). Those patients graded as having thicker skin were selected for an acid peel before surgery. Preoperative and 6-month postoperative rhinoplasty outcome evaluation (ROE) score were collected to assess outcomes as these are a validated patient reported outcome measure. Pearson chi-square test was used to determine statistically significance of the postoperative groups.

**Results:**

All patients with thick skin (grade 3) or above were offered a preoperative skin acid peel. A total of 42 patients received this peel. There was a mean improvement of 6.4 of the nose outcomes in patients who had *no* peel. The mean nose improvement in patients receiving the peel was 6.6 (*p* < 0.001). This indicates a marginal improvement of outcome in patients who had an acid peel before rhinoplasty.

**Conclusions:**

This nasal skin thickness scale provides a simple framework for classifying nasal skin thickness. Skin thickness helps us identify patients who would benefit from optimisation of their skin before surgery to get the best results. In this case optimisation of the skin was undertaken with chemical peels. The acid peel helped shrink the skin and improve outcomes after rhinoplasty. This classification helps select patients who have thick skin and can be warned about swelling, lack of definition and the possible need for preconditioning.

## Introduction

Undertaking rhinoplasty in patients with thick skin is a significant challenge as the results can be unpredictable. This is the first study to examine the effects of stratifying patients according to the thickness of their skin before plastic surgery. Practically speaking the benefits of this classification is applied to identifying patients who would benefit from preconditioning skin treatments as well as surgical manoeuvres to optimise their outcomes. TCA peels have a proven efficacy in optimizing skin and has been used routinely in plastic surgery to treat rhytids for many years. Traditional peels have used trichloroacetic acid or Croton oil in a preprepared formula with an indicator solution to measure depth of penetration. Courses are required to get training in this acid peel technique to avoid deep burns and scarring.^1^

Surgery is just one aspect of achieving patient satisfaction. Independent clinics routinely refer patients in house for a range of non-surgical treatments to get the best result from plastic surgery. Preconditioning is the safest way to treat thick skin rather than undertaking surgical manoeuvres such as thinning the skin in theatre which can risk necrosis of the skin. Given the importance of skin, it is remarkable that many plastic surgeons do not combine plastic surgery with skin treatments. Current skin treatments used routinely before and after surgery include acid peels, Morpheus8, lymphatic drainage and laser treatments.^1^^,^^2^ Combination treatment delivers the best results and this study aims to provide some structure to stratify which patients will benefit the most.

### Factors influencing nasal skin thickness

Patient age, ultraviolet (UV) exposure, genetics and prior trauma are factors which should be considered during the planning stage of rhinoplasty, due to their influence on the topography of the Soft Tissue Envelope (STE). Increased patient age is associated with decreased keratinocyte turnover, reducing its healing potential, as well as thinning of the dermis.^3^ The dermis of Asian and Middle Eastern patients has more numerous collagen fibres and larger fibroblasts, which explains its greater skin thickness.^1^^,^^2^ Collagen fibres are stacked more closely with ground substance in black skin compared to white skin.^4^ These differences between skin types are difficult to navigate, in Asian or Middle-eastern patients wishing for western shaped nose after rhinoplasty. The stratum corneum is comparable between skin types.^4^

### Skin thickness and rhinoplasty

Overall, the thickness of the STE can have a large impact on the overall cosmetic outcome of the procedure.^5^^,^^6^ Patients with a thinner STE are more likely to have visible irregularities in the osseocartilaginous framework, such as a tip bossae, as well as surgical adjuncts such as grafts and sutures.^7^, ^8^, ^9^ Conversely, a thicker envelope is at particular risk of tip deprojection, as well as more pronounced post-operative oedema.^10^, ^11^, ^12^ The thickness of the soft tissues are more associated with certain ethnicities.^13^, ^14^, ^15^

Thicker skin is associated with patients of African, Asian, and Middle Eastern backgrounds, who have additional reconstructive considerations in that there is a tendency for weaker cartilaginous support, making a defined tip even more technically challenging.^5^^,^^6^^,^^11^^,^^16^, ^17^, ^18^, ^19^, ^20^ Methods of optimising postoperative results in patients with a thicker STE have included intraoperative techniques such as thinning, altering the dissection plane as well as perioperative adjuncts such as skin contour sutures.^21^, ^22^, ^23^, ^24^ Perioperative strategies also include oral or intradermal corticosteroid injections, chemical peels such as Trichloroacetic acid (TCA), and oral and topical isotretinoin.^25^, ^26^, ^27^ An accurate preoperative assessment of the nasal STE thickness is therefore crucial in operative planning, counselling patients, and in expectation management. Although computer tomography (CT) and ultrasound imaging can be used to assess the soft tissues, in practice most surgeons rely on clinical examination alone.^28^ There is evidently a broad range of practice in optimising the soft tissue envelope, with no clear superior technique, and no means of standardising outcome measures.

To our knowledge, there is also no standardised tool for assessing the nasal soft tissues preoperatively. We therefore propose a photo-numerical scale for classifying the nasal STE thickness prior to rhinoplasty. We have illustrated the application of this scale by grading patients and allocating thicker STE grades into receiving preoperative TCA peels, as part of the standard practice of the senior author (R.U.). The scale does not correlate with the Fitzpatrick skin colour scale which relates to the response to UV exposure.

## Methods

### Development of the scale

A review of the current literature of MEDLINE by means of the OVID, PubMed, and Cochrane databases using the search terms (rhinoplasty) AND (scale OR skin thickness OR soft tissue envelope). No previous rhinoplasty skin thickness scales were identified. Relevant demographic data, including age, sex, ethnicity, medical comorbidities, smoking history and previous rhinoplasties were collected.

An expert panel consisting of six certified rhinoplasty surgeons discussed cutaneous features that they considered to be significant on clinical examination of the STE preoperatively. Based on these features, a Nasal Skin Thickness Scale (NSTS) was then formulated to grade patients from thin to a very thick on the STE based on clinical examination alone [Table 1, Table 2].

**Table 1:** NSTS grades according to clinical features.

NSTS grade	Skin assessment	Visual assessment	Palpation
1	Thin skin	Pale, translucent, atrophied skin appearance. Fine wrinkling. Subcuticular vessels or telangiectasia visible. Senile purpura and ecchymoses. Atrophied old healed scars. Most of nasal framework visible.	Smooth surface texture. Skin laxity. May feel dry. Skin thin on pinch test
2	Thin-medium skin	Some elements of framework visible	Skin thin on pinch test
3	Medium thickness skin	No visible framework	Sebaceous skin, or hair. and skin not thin on pinch test
4	Thick skin	No visible framework	Thick skin on pinch test, with sebaceous skin and hairs
5	Very thick skin	May reflect the light, have prominent follicles, nodular nasal contour. Inability to visualize deep vessels, superficial surface vessels may be present. No surface wrinkles.	Rough, leathery surface texture. Elasticity when skin is pulled taught, with recoil thereafter. May feel greasy. Thickness notable on pinch test.

86 % of the patients in this study had skin thickness grade 2 to 4. Grade 1 skin which is extremely thin and grade 5 skin which is equivalent to rhinophyma, are rare in the population and in this study. This grading system was used to identify patients with thick skin of grade 3 or 4 who would benefit from TCA peel preconditioning before their rhinoplasty.

**Table 2:** Clinical photography illustrating NSTS grades according to clinical features.

NSTS grade	Clinical examples
1	
2	
3	
4	
5	

Grade one is the thinner scale possible of the nose and grade 5 is the thick of skin indicating rhinophyma. The majority of patients in this study have a skin thickness of 2 to 4 NSTS. Rhinophyma which is classified as grade 5 in the scale is relatively rare. Extremely thin skin with minimal sebaceous glands and a visible framework is also relatively rare (grade 1 NSTS).

Thin skin on the nose tends to show the framework underneath while thick skin hides the framework. When the skin is pinched, thin skin lifts up easily while thick skin is sebaceous and hard to lift. These simple factors were used to score the patient’s skin thickness.

### Patient selection

A total of 166 patients were included in this study prospectively and graded by a board certified senior surgeon (R.U) between *June 2015* and *September 2018.*

All patients were graded according to the NSTS by the primary operating surgeon and fellow.

The aim of the study was to select patients who had thick skin and offer treatment with skin preconditioning to optimise the results of their rhinoplasty. None of the patients in this study underwent any surgical thinning of the skin.

Fifty patients were identified as having thick skin and so were candidates for preoperative skin optimisation with TCA peel [Table 3]. Patients classified with thin skin were not offered any preconditioning of their skin.

**Table 3:** Demographics table.

	Total	Total patients not receiving TCA peel (grades 1–5)	Thin skin patients not receiving TCA (grades 1–2) as not indicated
Number (%)	166 (100)	124 (75)	116 (70)
Age	33 (18–80)	36 (18–80)	36 (21–62)
Female (%)	120 (72)	99 (79)	93 (56)
NSTS (range)	2.2 (1–5)	1.8 (1–4)	1.8 (1–3)
Pre op ROE (range)	6.5 (3–13)	4.5 (4–12)	6.8 (3–13)
Post op ROE (range)	11 (10–18)	11 (10–18)	13.4 (11–17)
Mean ROE improvement	6.5	6.4	6.6

This table demonstrates the total number of patients who had thick and thin skin before rhinoplasty. Patients who had thick skin of a grade 4 or 5 NSTS were offered a TCA peel for preconditioning to optimise thick skin. The table demonstrates the patients who had thin skin and were not offered a TCA peel as this was not deemed to be beneficial for them. The majority of patients had thin skin and did not require a TCA peel for preconditioning. Only 30 % of the patients in this study were graded as having thick skin and so were eligible for a TCA peel before rhinoplasty. Ninety six percent of patients were satisfied with their rhinoplasty surgery went evaluated with the Rhinoplasty Outcome Evaluation (ROE) scoring system.

Eight patients declined the peel for personal reasons having reflected on the risks and benefits, of these, seven were grade 3 and one patient was a grade 4 NSTS [Table 4].

**Table 4:** This table demonstrates that 25 % of the patients in the study who had thick skin had a TCA peel before their rhinoplasty.

	Thick skin patients grades 3, 4 and 5 having TCA peel	Thick skin patients grades 3, 4 and 5 who declined a TCA peel
Number	42 (25)	8
Age (range)	31 (21–62)	33 (16–55)
Female (%)	21 (50)	6 (75)
NSTS (range)	3.3 (3–5)	3 (3–4)
Pre op ROE (range)	8.5 (3–13)	6 (6–10)
Post op ROE (range)	13.5 (11–17)	11 (10–14)
Average ROE improvement	6.8	4.8

The Rhinoplasty Outcome Evaluation (ROE) indicated that these patients were satisfied after their rhinoplasty procedure. When compared to patients who declined a TCA peel, the Rhinoplasty outcome evaluation indicates that patients were satisfied but this was not as much as the patients who had indeed proceeded with a TCA peel beforehand. This provides the evidence to demonstrate that patients who had thick skin, benefit from a TCA peel before rhinoplasty.

The skin thickness grading was performed on the second visit to clinic, after the patient had agreed to the rhinoplasty procedure and had sufficient time to consider the risks of surgery. A re-evaluation of the NSTS grade was performed on the day of surgery. The grading was performed on clinical examination alone. No other invasive or non-invasive assessments of the STE were conducted. The grading required an assessment of the appearance of the nasal skin as well as palpation of the skin and a pinch assessment.

All consenting patients over the age of 18 who underwent a primary open rhinoplasty were included within this study. Exclusion criteria included previous rhinoplasty, any scars on the nose and any previous non-surgical treatments such as hyaluronic acid filler or ‘non-surgical rhinoplasty’.

The patients were not using any Isotretinoin nor other skin preconditioning before being entered into the study. We only found two patients who had very thick grade 5 skin and they were referred to a dermatologist.

This study adheres to the standards of the declaration of Helsinki and complies with the Health Insurance Portability and Accountability Act of 1996. Data was collated in a standardised format using Microsoft Office Excel 2019.

### Rhinoplasty technique and perioperative care

All patients underwent open rhinoplasty with standard perioperative care as per the protocol of the senior author. All patients completed Rhinoplasty Outcome Evaluation (ROE) scores before and 12 months after surgery and verified by an independent clinician to that operating. The ROE score is a 6-point questionnaire aimed to subjectively assess patient satisfaction with their nose.

**Fig. 1:**
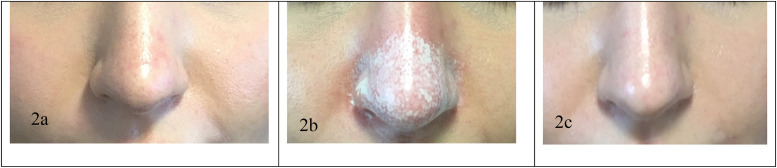
Clinical photographs illustrating before application of the 15 % TCA acid peel (a), peel ‘frosting’ (b) and 12 months following the peel (c). The patient reported that the TCA peel has reduced the oily nature of the tip of the nose. The patient also reported that the acid peel has reduced the size of the tip in a subtle way. There is no numbness nor pigmentation associated with this procedure. Pigmentation can occur in dark skinned individuals especially if sunscreen is not applied diligently. Patients are reassured that any pigmentation resolves over 3 to 6 months with the regular use of sunscreen. The exact procedure is shown in the video attached to this article.

As part of his standard practice, the senior author routinely uses TCA peel preoperatively for patients he deemed as having thicker soft tissues. To illustrate the application of the NSTS, we considered thicker skin as grades 3, 4 and 5 on the NSTS. All those graded as NSTS 3 or above were offered preoperative TCA peel. A 15 % TCA formulation was applied until a 'white frosting’ appeared, indicating adequate penetration into the dermis whilst avoiding a chemical burn [Fig. 1]. Patients receive the peel no later than 4 weeks prior to surgery.

### Statistical analysis

Pearson chi-square test was used to determine statistically significance of the two postoperative groups. A two-tailed paired samples *t*-test was used to ascertain differences within groups of means and standard error. Statistical significance was determined by a *P* value of <0.05. Microsoft Excel (Microsoft Corp., Redmond, Wash.) and SPSS Statistics for Windows, version 27.0 (IBM Corp, Armonk, N.Y.) were used to calculate averages, percentages, SDs, and *P* values.

Comparisons with a *P* value <0.05 were considered statistically significant. Based on an alpha of 0.05, power analysis estimates that significant differences will be detected between groups with a sample size of 41 people in each group with a power exceeding 90 %.

## Results

### Study demographics

**Fig. 2:**
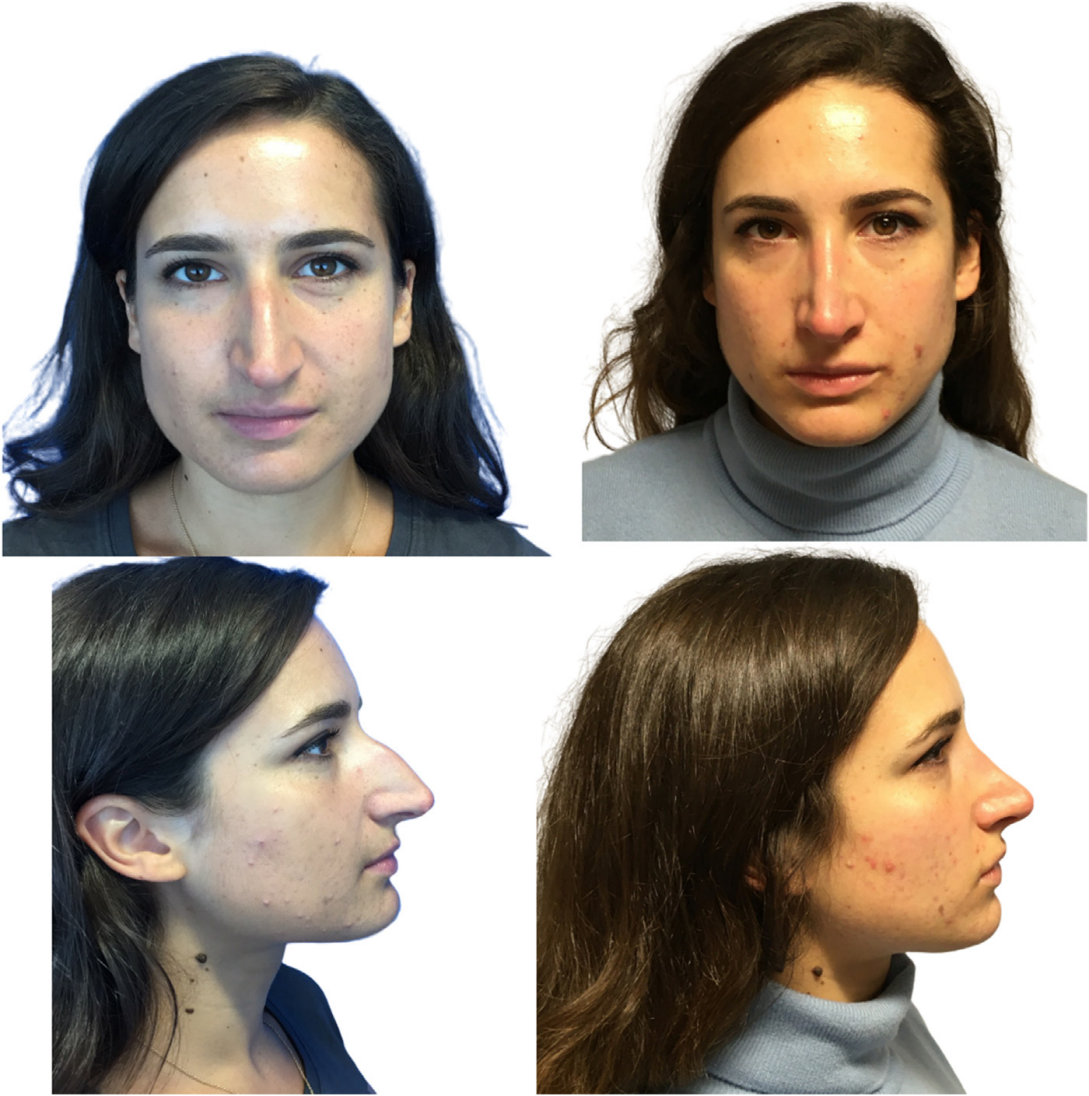
This is an image of a 26-year-old patient, 12 months after surgery who had grade 2 skin which is relatively thin. She was therefore not a candidate for a TCA peel and did not have this preconditioning which only delivers a benefit to patients with thick skin. Her ROE score assessment was 9 before surgery and increased to 13 after surgery indicating that she was satisfied with the results of her rhinoplasty.

**Fig. 3:**
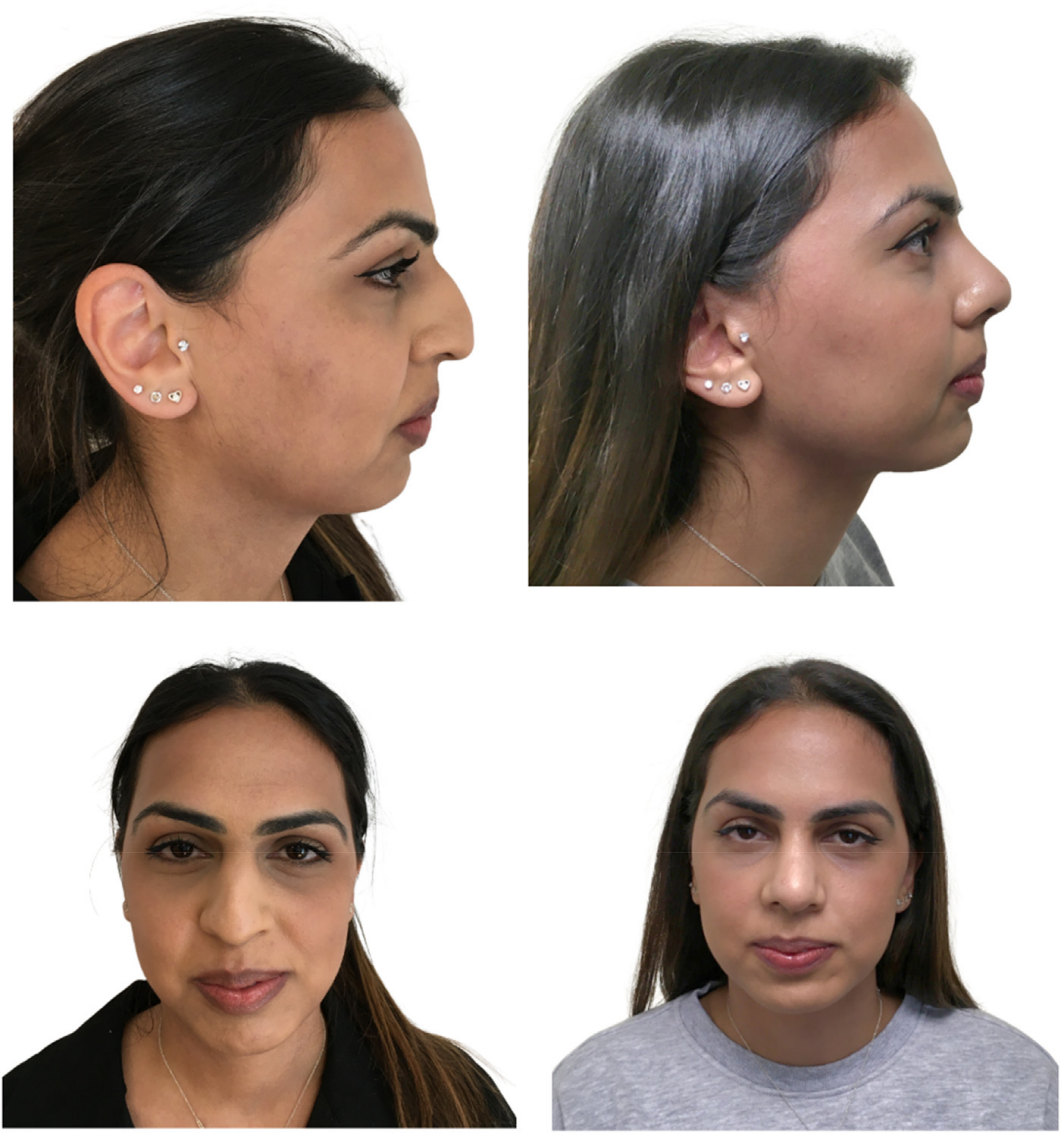
Preoperative and postoperative views at 12 months of a 28-year-old patient with Grade 3 nasal skin thickness who declined a TCA peel prior to surgery. The patient was satisfied with her rhinoplasty. Her preoperative ROE score was 8 and this increased to 14 after surgery. However, the authors feel that a TCA peel would have improved the definition of her tip. Patients with thick skin benefit from preconditioning of their nose tip skin with an acid peel to maximise the benefit from the support struts and sutures applied to the lower lateral cartilages during rhinoplasty.

A total of 166 patients were included in this study. The average postoperative follow up time was 20 months. All postoperative images were taken at a minimum of 12 months. The total population consisted of 46 males and 120 females, and mean average age was 33 years (range 18–80). Demographics of the population can be found in Table 2. All the patients underwent primary cosmetic rhinoplasty. Examples of postoperative outcome are illustrated in Fig. 2, Fig. 3. Revision rhinoplasty cases for both cosmetic or reconstructive indications were excluded. Patients were then categorised into NSTS grade. Those with a grade 3 or above were offered TCA peel as per standard management of thicker STE according to the senior author (R.U.).

### TCA peel group with thick skin before rhinoplasty

Forty-two patients had a TCA peel before surgery and consisted of 11 male and 31 female patients. The mean average age was 31 (range 21–62), and mean Skin Thickness score was 3.3 (range 3–5). The mean improvement in pre and postoperative ROE was a 6.7(SD 2.52).

The eight patients who did not have a TCA peel preconditioning still had an improvement in their ROE score following surgery, with a mean improvement of 4.6 (SD 2.2) (Table 3).

Therefore, the patients who had a TCA peel before surgery clearly had a much better outcome than those without the TCA Peel.

One hundred and twenty-four patients underwent open primary rhinoplasty *without* a preoperative peel. The cohort consisted of 35 males and 89 female patients, the average age was 36 (range 18–80), and the mean NSTS score was 1.8 (range 1–4). The mean ROE improvement was 6.4 (*p* < 00.1).

No patients in either group had been reclassified into a different NSTS group on re-evaluations on the day of surgery. This indicates good inter-rater reliability of the skin thickness scoring system between different surgeons.

The data indicates that although patients with thick skin are known to get more swelling and scar tissue after rhinoplasty, they tend to be much happier with their results than thin-skinned patients.

## Discussion

The fundamental problem with pre-operative assessment of skin thickness is the lack of a standardized method for measurement. While most senior surgeons visually assess the framework, the lack of a reproducible objective limits the availability of comparative data. This study provides a framework for identifying patients with thicker skin and an algorithm for treatment. We demonstrate that accurate clinical assessment can predict the patients that may benefit from skin optimization perioperatively and can have thinning of skin during surgery in a safe manner without risking necrosis.

Assessing the skin first involves looking at area where it is thickest. On the nose the tip has the thickest skin. The surgeon currently will look for any open pores or sebaceous quality that will indicate thick skin. Consideration is also given to the visibility of the underlying framework. Skin is altered with time depending on the degree of exposure to sunlight. This important factor is incorporated into the scale to account for thin skin in some elderly patients.

Previous studies have used ultrasound and CT scanning for measurement of soft tissue thickness.^13^^,^^25^^,^^29^ Although these modalities can provide useful information about the soft tissues and osseocartilaginous components of the nose, in practice these entail additional costs, time, visits to clinic, as well as radiation exposure. Kosins and Obagi described the use of Skin pinch thickness at the nose keystone junction as a means of assessing the STE.23

Although a simple and pragmatic assessment, this “nasal pinch test” does not provide an accurate scale for clinical management of patients, nor for subsequent comparing of outcomes amongst similar patient groups.

We have incorporated this pinch test in our NSTS but added a visual clinical assessment of the tip, where the skin is thickest on the nose. Our scale also takes into consideration the adnexal structures of the skin such as hairs and the oiliness of the skin in order to help identify very thick nose skin.^22^

Using the scale in our own clinical practice has allowed us to analyse out own patient cohort and preferences such as the use of a TCA peel. Overall, our patient cohort benefited in undergoing rhinoplasty. Furthermore, those patients who were identified as having a thicker soft tissue envelope and received a preoperative TCA peel tended to see a greater improvement than those of thinner NSTS, and an even more marked improvement to those with high NSTS who declined the peel. We the authors fully acknowledge these conclusions are based on a small sample size but provides invaluable objective measures to assess our own practices.

Dey et al.^26^ found that the sebaceous quality of skin correlated well with thick nasal skin. Conversely, they found that a visible cartilaginous framework correlated well with thin nasal skin. We used both these parameters to define our clinical skin thickness measurement scale. That same study found that experienced rhinoplasty surgeons could identify thick nasal skin using a visual analogue score and this correlated well with imaging studies. This validates the use of a visual/clinical scale. Our scale therefore incorporates these findings with the published skin pinch test, to create a skin thickness scale that can be applied easily by all surgeons, irrespective of their experience. This scale brings us a step closer in making an objective assessment of the soft tissue envelope of the face.

To ensure that this scale is relevant for clinical practice, we have created an algorithm to identify who would benefit from treatment of thick skin which can compromise a good result after cosmetic surgery of the face. The main benefit of the scale is to select patients who may benefit from perioperative optimisation, such as acid peels, as well as offer a means of standardising outcomes. The authors maintain that in categorising patients thus, not only will it provide a comparison of perioperative techniques amongst similar patients but will also aid preoperative counselling. This will make a major contribution to the counselling and consent process before cosmetic surgery is undertaken.

## Conclusion

We present a simple, clinically orientated scale for assessing patients prior to rhinoplasty, and can be used to direct perioperative care, as illustrated by our preference for the application of TCA peels in thicker skinned individuals.

## Patient consent

Patient consent for publication of images was obtained for this publication.

## Funding

None.

## Ethical approval

Not required.

## Conflicts of interest

None declared.
